# *In vitro* cross-talk between metastasis-competent circulating tumor cells and platelets in colon cancer: a malicious association during the harsh journey in the blood

**DOI:** 10.3389/fcell.2023.1209846

**Published:** 2023-08-02

**Authors:** Zahra Eslami-S, Luis Enrique Cortés-Hernández, Ilias Glogovitis, Mafalda Antunes-Ferreira, Silvia D’Ambrosi, Keerthi Kurma, Françoise Garima, Laure Cayrefourcq, Myron G. Best, Danijela Koppers-Lalic, Thomas Wurdinger, Catherine Alix-Panabières

**Affiliations:** ^1^ Laboratory of Rare Circulating Human Cells—University Medical Center of Montpellier, Montpellier, France; ^2^ CREEC/CANECEV, MIVEGEC (CREES), Université de Montpellier, Centre National de la Recherche Scientifique, Institut de Recherche pour le Développement, Montpellier, France; ^3^ European Liquid Biopsy Society (ELBS), Hamburg, Germany; ^4^ Department of Neurosurgery, Amsterdam University Medical Centers, Vrije Universiteit Amsterdam, Amsterdam, Netherlands; ^5^ Cancer Center Amsterdam, Amsterdam University Medical Centers, Vrije Universiteit Amsterdam, Amsterdam, Netherlands; ^6^ Brain Tumor Center Amsterdam, Amsterdam University Medical Centers, Vrije Universiteit Amsterdam, Amsterdam, Netherlands; ^7^ Mathematical Institute, Leiden University, Leiden, Netherlands

**Keywords:** platelets, circulating tumor cells (CTCs), epithelial-to-mesenchymal transition (EMT), tumor-educated platelets (TEPs), cancer, metastasis

## Abstract

**Background:** Platelets are active players in hemostasis, coagulation and also tumorigenesis. The cross-talk between platelets and circulating tumor cells (CTCs) may have various pro-cancer effects, including promoting tumor growth, epithelial-mesenchymal transition (EMT), metastatic cell survival, adhesion, arrest and also pre-metastatic niche and metastasis formation**.** Interaction with CTCs might alter the platelet transcriptome. However, as CTCs are rare events, the cross-talk between CTCs and platelets is poorly understood. Here, we used our established colon CTC lines to investigate the colon CTC-platelet cross-talk *in vitro* and its impact on the behavior/phenotype of both cell types.

**Methods:** We exposed platelets isolated from healthy donors to thrombin (positive control) or to conditioned medium from three CTC lines from one patient with colon cancer and then we monitored the morphological and protein expression changes by microscopy and flow cytometry. We then analyzed the transcriptome by RNA-sequencing of platelets indirectly (presence of a Transwell insert) co-cultured with the three CTC lines. We also quantified by reverse transcription-quantitative PCR the expression of genes related to EMT and cancer development in CTCs after direct co-culture (no Transwell insert) with platelets.

**Results:** We observed morphological and transcriptomic changes in platelets upon exposure to CTC conditioned medium and indirect co-culture (secretome). Moreover, the expression levels of genes involved in EMT (*p* < 0.05) were decreased in CTCs co-cultured with platelets, but not of genes encoding mesenchymal markers (*FN1* and *SNAI2*). The expression levels of genes involved in cancer invasiveness (*MYC, VEGFB, IL33, PTGS2*, and *PTGER2*) were increased.

**Conclusion:** For the first time, we studied the CTC-platelet cross-talk using our unique colon CTC lines. Incubation with CTC conditioned medium led to platelet aggregation and activation, supporting the hypothesis that their interaction may contribute to preserve CTC integrity during their journey in the bloodstream. Moreover, co-culture with platelets influenced the expression of several genes involved in invasiveness and EMT maintenance in CTCs.

## 1 Introduction

Cancer progression and dissemination are complex processes. A key step in cancer dissemination (i.e., metastasis) is the survival of circulating tumor cells (CTCs) in the blood (Dujon et al., 2021). Indeed, CTCs that cannot overcome the physical and biological assaults in the blood (e.g., shear forces, anoikis, immune system activity) will not produce metastases (Eslami-S et al., 2020). CTC survival might be influenced by their capacity to adapt, leading to clonal selection, and also by their interaction with the various blood components. It has been suggested that CTC interaction with blood platelets might promote their survival (Ward et al., 2021).

The direct interactions between cancer cells and platelets in the bloodstream have been extensively described, including in patients. In 2012, Gil-Bernabé et al. (2012) showed that tissue factor triggers clot formation by platelets around tumor cells. They demonstrated that macrophage recruitment is required for cancer cell survival and also that coagulation is essential for the recruitment of a similar subset of monocytes/macrophages to distant sites where CTCs settle and prosper. Another study showed that the recruitment of granulocytes is not primarily due to tumor cell-derived signals, but relies on the platelet-derived CXCL5/7 chemokines from platelet–tumor cell microthrombi that promote metastasis (Labelle, Begum and Hynes, 2014). Moreover, through the cancer cell-platelet cross-talk, platelet receptors are expressed also on tumor cells that acquire a platelet-like phenotype (i.e., platelet mimicry). This allows tumor cells to evade the immune surveillance (Tímár et al., 2005). Moreover, through CTC-platelet interactions, activated platelets can transfer major histocompatibility complex-1 (MHC-1) components onto CTCs, thereby allowing CTCs to be recognized as normal cells by the immune system (Placke et al., 2012). Cancer has also been associated with a pro-thrombotic state (Fernandes et al., 2019). An *in vitro* study using an ovarian cancer cell line and platelets revealed significant platelet adherence and activation (based on P-selectin surface expression) and increased invasive potential of cancer cells (Cooke, Spillane, Sheils, O’Leary and Kenny, 2015). Most recently, it has been reported that in patients with cancer, the platelet transcriptome is modified and may represent a diagnostic tool (Best et al., 2017; Best et al., 2015; In ’t Veld et al., 2022; Sol et al., 2020). The reprogramming of the platelet RNA repertoire may be caused by multiple processes, including tumor-derived biomolecules and signals, such as extracellular vesicles and thrombin, alterations in the transcriptional programs of megakaryocytes (the platelet precursors), and mRNA splicing in mature platelets exposed to tumor cell factors (Heinmöller et al., 1996; Nilsson et al., 2011; Best et al., 2018; Davizon-Castillo, Rowley and Rondina, 2020). Consequently, such tumor-educated platelets are considered a potential liquid biopsy biomarker (Best et al., 2015).

Conversely, the specific changes associated with CTC-platelet interactions using CTC models are not well described. This is mainly explained by the fact that CTCs are rare events in the systemic circulation and this further complicates the study of CTC-platelet interactions in patient samples. Furthermore, establishing *in vitro* and *in vivo* CTC models is challenging and only few groups accomplished this challenge (Gao et al., 2014; Yu et al., 2014; Cayrefourcq et al., 2015; Khoo et al., 2016). We have successfully established an unique and permanent series of colon CTC lines from a patient with unresectable colorectal adenocarcinoma, abdominal and mediastinal lymph node invasion and liver metastases (Cayrefourcq et al., 2015, 2021; Soler, Cayrefourcq, Mazel and Alix-Panabieres, 2017). Here, we used these cell lines to study CTC-platelet interactions *in vitro*. Our objectives were 1) to determine CTC impact on platelet behavior and protein expression; 2) to compare the gene expression profiles of platelets before and after indirect (presence of a Transwell) co-culture with the three colon CTC lines; and 3) to determine the effect of direct co-culture (no Transwell insert) with platelets on CTC morphology (e.g., epithelial-mesenchymal transition, EMT) and expression of genes involved in the metastatic process.

## 2 Material and methods

### 2.1 Platelet isolation

Blood samples from independent healthy donors, who had not taken any drugs known to affect platelet function, were collected by venipuncture (21-gauge butterfly needle without tourniquet) in 6 mL purple-cap BD vacutainers containing the anti-coagulant EDTA (BD-Plymouth ref: 367864A) at the Établissement Français du Sang (EFS). Deidentified blood samples were processed at the University Medical Center of Montpellier, France. This study used blood samples from the EFS and ethics approval was not needed according to the French legislation as blood samples were volunteering donated and all donors were aware that blood collection will be used for research purposes. To minimize the effects of long-term platelet storage at room temperature (loss of RNA quality/quantity and platelet activation), blood was collected and processed on the same day. Platelets were isolated from whole blood with a two-step differential centrifugation protocol. Before each centrifugation step, 400 nM prostaglandin I2 (Sigma-Aldrich ref: P6188) was added to prevent platelet activation. Briefly, platelet-rich plasma was prepared by centrifugation at 120 g, at room temperature, for 20 min. To avoid white blood cell contamination, only 2/3 of the plasma was collected for the second centrifugation step (360 g for 20 min) to pellet platelets. Then, platelets were washed with Hanks’ Balanced Salt Solution supplemented with 4 mM EDTA and 0.35% bovine serum albumin. Before each experiment, platelet purity was assessed by microscopic visualization (1–5 nucleated cells per 10 million platelets), as previously described (Rolf et al., 2005; Best et al., 2015), to limit confounding factors (e.g., presence of many white blood cells).

### 2.2 CTC lines

Three permanent CTC lines (CTC-MCC-41, CTC-MCC-41.4, and CTC-MCC- 41.5G) obtained by our group from a 57-year-old man with metastatic colon cancer (Cayrefourcq et al., 2015, 2021; Soler et al., 2018) were used. The first line (CTC-MCC-41) was established using a blood sample collected before treatment initiation with the 5-fluorouracil-irinotecan (FOLFIRI) combination and bevacizumab (5 cycles). Due to biological progression after the fifth FOLFIRI cycle, second-line treatment was initiated with 5-fluorouracil-oxaliplatin (FOLXFOX) and bevacizumab, but was stopped after the third cycle because of disease progression when the second line was obtained (CTC-MCC-41.4). The patient died about 6 months after diagnosis due to cancer progression. The third line (CTC-MCC-41.5G) was derived from a blood sample obtained 1 week before the patient’s death. Their phenotypic and molecular characterization indicated that they present common features and all display epithelial-to-mesenchymal plasticity and stem cell-like characteristics (Alix-Panabières et al., 2017; Soler et al., 2018). They also represent a unique biological material to study clonal evolution during therapy and disease progression (Cayrefourcq et al., 2021).

CTC lines were grown in suspension in standard conditions using RPMI 1640 (Eurobio ref: CM1RPM00-01) supplemented with 10% fetal calf serum (Eurobio ref: CVFSVF00-01), insulin-transferrin-selenium (Gibco ref: 51300-044), L-glutamine (Eurobio ref: CSTGLU00-0U), epidermal growth factor (Miltenyi Biotec ref: 130-093-825), and beta fibroblast growth factor (Miltenyi Biotec ref: 130-093-840). They were maintained in a cell culture incubator at 37°C and 5% CO_2_. To ensure that the CTC lines maintained their original features, many stocks of each CTC line at very early passages (2–5), were stored in liquid nitrogen. All experiments were performed using cells at these very first passages. Before platelets were added, CTCs were cultured in serum-free medium (conditioned medium) for 24 h. The control serum-free medium was incubated for the same time as the CTCs.

### 2.3 Transient co-culture of CTCs and platelets

CTCs were seeded the day before platelet isolation and incubated in serum-free medium overnight. Then, they were co-cultured with 150,000 platelets/μL for 24 h, as recommended (Labelle, Begum and Hynes, 2011a; Zhang et al., 2019; Plantureux et al., 2020). Freshly isolated platelets from the same donor were collected immediately after isolation as control at 0 h. For platelet RNA sequencing (RNA-seq) experiments, a Transwell insert with a pore size of 0.4 µm (Corning^®^ ref: 3450) was placed on the plates to avoid mixing CTCs and platelets, but to allow platelet exposure to the CTC secretome (i.e., indirect co-culture). After 24 h, CTCs (see 2.10) and platelets were collected. Platelets were spun and resuspended in RNAlater (Invitrogen, Thermo Fisher Scientific ref:01025060) for transcriptomic analysis. Thrombin (Sigma Aldrich ref: 10602400001) was used (0.5 unit/well) as positive control of platelet activation. Untreated CTCs (without platelets) were used as negative control.

### 2.4 Microscopy analysis

Platelet morphology was assessed with a conventional fluorescent inverted microscope. Bright-field micrographs of representative areas of platelet cultures in 6-well plates were used for the analysis.

### 2.5 Flow cytometry

FACS buffer (phosphate-buffered saline with 2% fetal bovine serum and 2 mM EDTA) was used for staining and washing cells. For platelet immunostaining, anti-CD62P-FITC (Beckman Coulter ref: A07790) and anti-CD41/61-PE (Miltenyi Biotec ref: 130-109-424) antibodies were used.

To determine platelet effects on EMT induction in the three colon CTC lines, cells were fixed before permeabilization with the IntraPerp Permeabilization kit (Beckman Coulter ref: A07803). Then, they were incubated with anti-cytokeratin 19 (CK19) Alexa Fluor^®^ 488 (Exbio ref: A4-120-C100) and anti-EpCAM-APC (Miltenyi Biotec ref:130-091-254) antibodies followed by analysis using a Gallios flow cytometer (Beckman Coulter, Brea, CA, United States) at the Rio-Imaging Cytometry Core facility. Flow cytometry data were analyzed with the Kaluza software (Beckman Coulter Life Sciences).

### 2.6 RNA isolation, cDNA library construction, and platelet RNA-sequencing

Total RNA was isolated from platelets using the mirVana miRNA isolation kit (Ambion, Thermo Scientific ref: AM1560), according to the manufacturer’s instructions. RNA quality and quantity were assessed with RNA 6000 Picochip (Bioanalyzer 2100, Agilent, Santa Clara, CA, United States). Only platelet RNA samples with a RIN value > 7 and/or distinctive rRNA curves were included for analysis.

cDNA libraries were generated using 500 ng of total platelet RNA as input. The SMARTer kit V3 Stranded Total RNA-seq kit (Pico Input Mammalian User Manual, TakaraBio, Kyoto, Japan) was used for library preparation. After addition of Illumina adapters and the final PCR amplification for 16 cycles, libraries were purified with Agencourt AMPure XP beads (Beckman Coulter ref: A63881) (Best et al., 2019).

Single-end sequencing (2 × 100 bp) was performed on an Illumina HiSeq 4000 device using the TruSeq reagents, version 4. Index combinations were chosen according to the Illumina recommendations.

### 2.7 Platelet RNA-sequencing data processing in Galaxy

FASTQ files obtained by RNA-seq were analyzed with Galaxy Docker Image (version 20.09) and R (version 4.0.1). Pre-processing, trimming, mapping, and mRNA count quantification were performed with tools available via Galaxy Docker Image (Afgan et al., 2018). The pre-processing step included the concatenation of the technical replicates for each sample and was performed with the Concatenate datasets tail-to-head tool (version 0.1.1). The read quality of FASTQ files was checked with the FastQC (version 0.72) tool and adapters were removed with TrimGalore (version 0.6.7). Reads that passed the quality filtering procedure were mapped to the *Homo sapiens* genome assembly (GRCh38.103) with the Hisat2 (version 2.1.0) alignment tool (Kim, Langmead and Salzberg, 2015) and transcript counts were extracted with FeatureCounts (version 2.0.1) (Liao, Smyth and Shi, 2014).

### 2.8 Data processing in R

Transcript with low count number were filtered out with the filterByExpr-function from the EdgeR-package (version 3.38.1) (Robinson, McCarthy and Smyth, 2010). Differential expression analysis was performed with the EdgeR package using Relative Log Expression as normalization method in R (version 4.0.1). The most significant differentially expressed transcripts were filtered based on the following criteria: |log2FC| > 1 and *p*-value <0.05. Volcano transcripts plots and heatmaps were generated with the EnhancedVolcano package (Blighe, Rana and Lewis, 2019) and the Pheatmap function (Kolde, 2012), respectively.

### 2.9 Functional pathway enrichment

To investigate the role of the differentially expressed mRNAs, a gene set enrichment analysis (GSEA) was performed with the GSEA software using the “hallmark” gene set collection in the Molecular Signature Database (MSigDB) (Subramanian et al., 2005) (version 7.5.1). A compendium of Gene Ontology terms (biological processes, molecular functions and cellular components) and a KEGG pathway analysis were carried out with the goseq tool (Young, Wakefield, Smyth and Oshlack, 2010) (version 1.44.0) using Galaxy.

### 2.10 Gene expression in CTC lines

After direct co-culture (no Transwell insert) for 24 h, CTCs were collected, and total RNA was extracted using the RNeasy Plus Mini Kit (QIAGEN ref:74134) according to the manufacturer’s instructions. RNA concentration and purity were determined using a NanoDrop 2000c spectrophotometer (Thermo Fisher Scientific, Waltham, United States) and RNA was converted to complementary DNA (cDNA) using SuperScript III First-Strand (Invitrogen ref:18080-400). Changes in the expression of EMT-related genes and of key genes in the metastatic process were monitored using semi-quantitative PCR and glyceraldehyde-3-phosphate dehydrogenase (*GAPDH*) as control. PCR reactions were performed with a QuantStudio 5 real-time PCR system (Thermo Fisher Scientific, Waltham, United States) and the Brilliant III Ultra-Fast SYBR^®^ Green Master Mix (Agilent Technologies ref: 600882). The relative fold-change expression of mRNAs was calculated with the 2^−ΔΔCT^ method**.** The expression level of each gene was analyzed in duplicate and in three independent experiments (N = 3). The primer list is in Table 1.

**TABLE 1:** Sequences of the forward and reverse primers used for RT-qPCR.

Human genes	Primer sequence
*GAPDH*	Forward: GTC​TCC​TCT​GAC​TTC​AAC​AGC​G
	Reverse: ACCACCCTGTTECTGTAGCCAA
*TGFß2*	Forward: AAG​AAG​CGT​GCT​TTG​GAT​GCG​G
	Reverse: ATG​CTC​CAG​CAC​AGA​AGT​TGG​C
*EPCAM*	Forward: ACA​AGG​ACA​CTG​AAA​TAA​CCT​GC
	Reverse: TTT​TGA​GAA​GAA​TTT​TGA​ACC​AGA​T
*CK19*	Forward: GCG​ACT​ACA​GCC​ACT​ACT​ACA​CG
	Reverse: CTT​CGC​ATG​TCA​CTC​AGG​ATC​T
*CDH1*	Forward: CCGAGAGCTACACGTTC
	Reverse: TCT​TCA​AAA​TTC​ACT​CTG​CC
*FN1*	Forward: ACA​ACA​CCG​AGG​TGA​CTG​AGA​C
	Reverse: GGA​CAC​AAC​GAT​GCT​TCC​TGA​G
*SNAI2*	Forward: CCT​TCC​GTA​ACT​GGA​TGA​ACT​C
	Reverse: GGA​TGC​TTC​CCT​AAT​TCA​ACA​G
*MYC*	Forward: GCT​GCT​TAG​ACG​CTG​GAT​TT
	Reverse: GAG​TCG​TAG​TCG​AGG​TCA​TAG​TT
*VEGFB*	Forward: AAG​GAC​AGT​GCT​GTG​AAG​CCA​G
	Reverse: TGG​AGT​GGG​ATG​GGT​GAT​GTC​A
*IL33*	Forward: GCC​TGT​CAA​CAG​CAG​TCT​ACT​G
	Reverse: TGT​GCT​TAG​AGA​AGC​AAG​ATA​CTC
*PTGS2*	Forward: CGG​TGA​AAC​TCT​GGC​TAG​ACA​G
	Reverse: GCA​AAC​CGT​AGA​TGC​TCA​GGG​A
*PTGER2*	Forward: GAC​CAC​CTC​ATT​CTC​CTG​GCT​A
	Forward: AAC​CTA​AGA​GCT​TGG​AGG​TCC​C

### 2.11 Statistics

Statistical analyses were performed using Prism 8 (GraphPad Software Inc., San Diego, CA, United States). All experimental data were compared with an independent two-tailed unpaired *t*-test. Data are presented as mean values ±standard error of the mean (SEM). *p*-values <0.05 were considered significant.

## 3 Results

### 3.1 Platelet morphological changes and activation upon exposure to CTC conditioned medium

**FIGURE 1:**
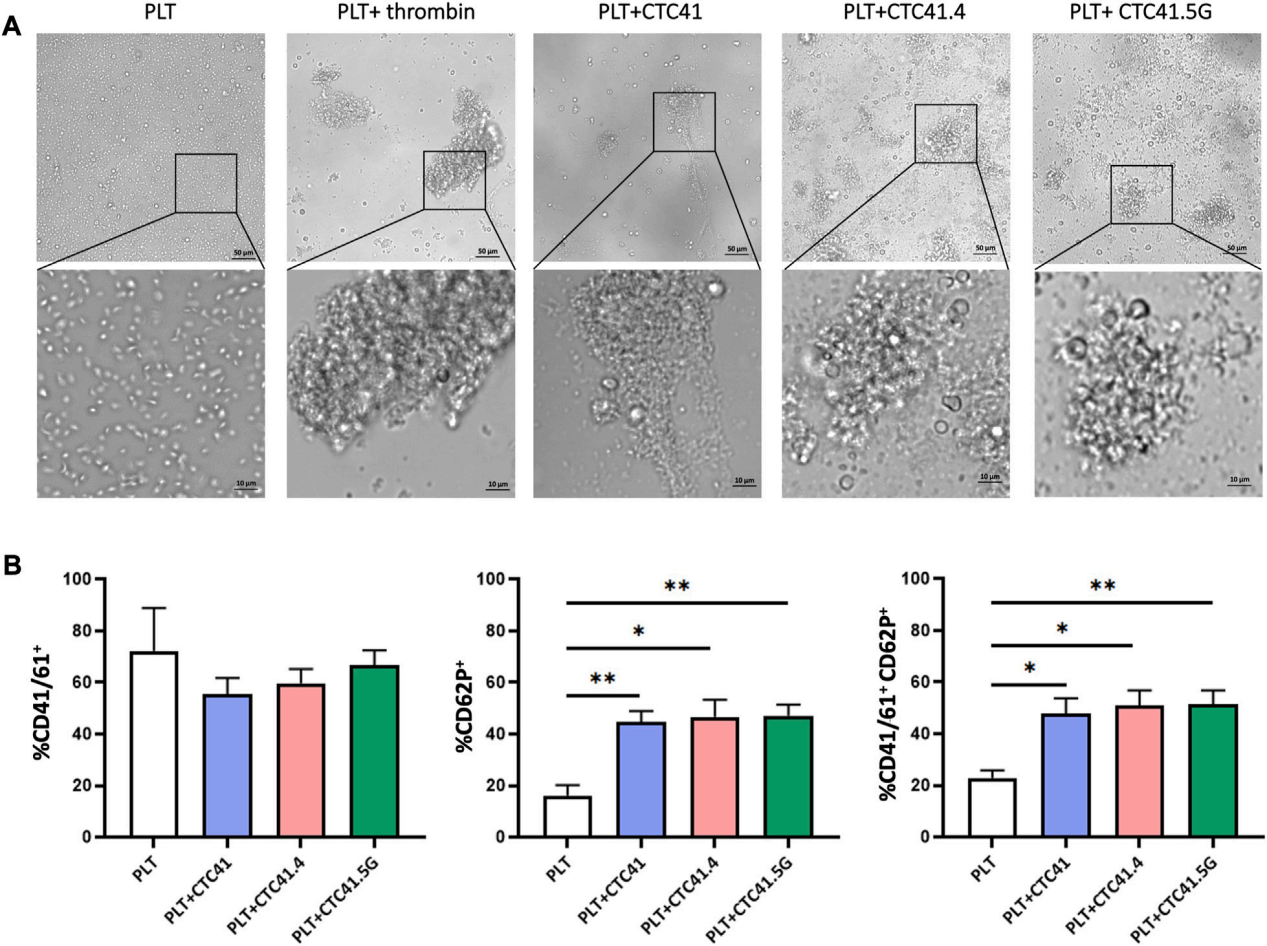
Platelet morphological changes after exposure to conditioned medium from three CTC lines. **(A)** Representative images (×20 magnification—zoom ×40 magnification) of the morphological changes and aggregation of platelets (PLT) incubated with conditioned medium from three colon CTC lines (CTC41, CTC41.4, and CTC41.5G), with control medium, and with thrombin (positive control). **(B)** Quantification of CD41/61^+^, CD62P^+^, and CD41/61^+^CD62P^+^ platelets in the different culture conditions described in **(A)** by flow cytometry. Values are the mean ± SEM, *n* = 3/ group; *p < 0.05, **p < 0.005 vs. control (PLT) (unpaired t-test).

First, we evaluated platelet aggregation by incubating platelets with conditioned medium from the three colon CTC lines (CTC41, CTC41.4, and CTC41.5G), control medium, or thrombin (positive control). After 24 h of incubation, we observed changes in platelet shape and aggregation. Compared with platelets exposed to control medium, in which the oval shape morphology was preserved, we observed the formation of aggregates (clot formation) in platelets exposed to CTC conditioned medium and to thrombin (Figure 1A). To fully characterize platelet activation, we analyzed platelets by flow cytometry. The results revealed changes in the expression of surface proteins associated with platelet activation, such as CD62P and CD41/61 (Supplementary Figure S1). Specifically, the percentage of CD62P^+^ platelets was significantly increased in samples exposed to conditioned medium from CTC41 (*p = 0.008*), CTC41.4 (*p = 0.016*), and CTC41.5G (*p = 0.006*) cells compared with control (medium only). Similarly, the percentage of CD41/61^+^CD62P^+^ platelets was significantly increased upon exposure to conditioned medium from CTC41 (*p = 0.018*), CTC41.4 (*p = 0.011*), and CTC41.5G (*p = 0.009*) cells compared with medium alone **(** Figure 1B **)**. Although the activation observed after treatment with CTC conditioned medium was not significant compared with the untreated condition, all three CTC lines caused platelet activation, suggesting that CTC lines secrete specific factors (e.g., vesicles, proteins, RNAs, metabolites) that can induce platelet activation. These findings demonstrate that aggressive cancer cell-platelet interaction is a universal phenomenon; however, the degree of physical contact between platelets and cancer cells and the subsequent platelet activation need to be better investigated.

### 3.2 Transcriptomic changes in platelets upon indirect co-culture with the three CTC lines

**FIGURE 2:**
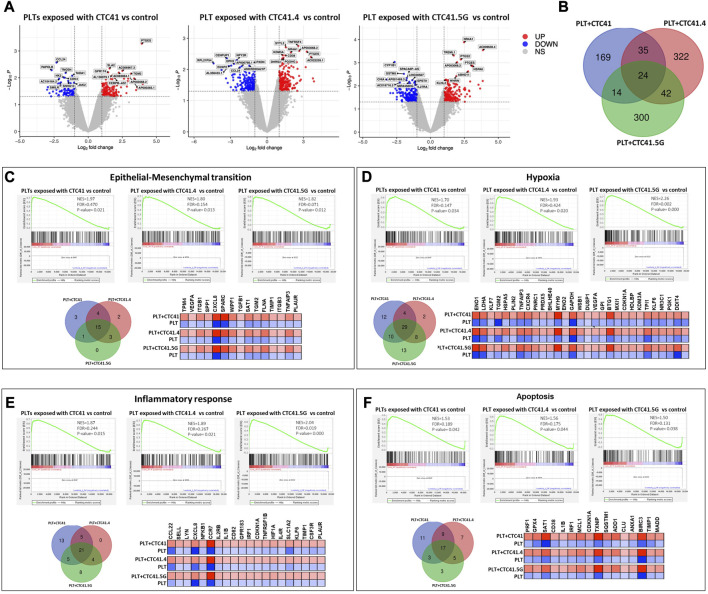
Platelet transcriptomic changes upon indirect co-culture with three CTC lines. **(A)** Volcano plots showing the differential gene expression analysis of RNA-seq data. The log-transformed *p*-values are plotted on the *y*-axis and the log2 fold change values of platelets after indirect co-culture with CTC41, CTC41.4, and CTC41.5G cells vs*.* control (platelets alone) are plotted on the *x*-axis. The criteria were relative expression fold change ≤ −2.0 or ≥2.0 and *p* ≤ 0.05. Red dots and blue dots represent increased and decreased RNAs, respectively. **(B)** Venn diagram showing the number of shared differentially expressed mRNAs in platelets after indirect co-culture with CTC41, CTC41.4, and CTC41.5G cells vs*.* control. **(C–F)** Gene Set Enrichment Analysis (GSEA) was performed using the RNA-seq data to compare the transcriptome profiles of platelets after indirect co-culture with CTC41, CTC41.4, and CTC41.5G cells vs*.* control. **(C,D)** GSEA results showing significant enrichment of the EMT and Hypoxia signaling pathways among mRNAs increased in platelets after indirect co-culture with CTC41, CTC41.4, and CTC41.5G cells vs*.* control. **(E,F)** GSEA results showing that the Inflammatory response and Apoptosis pathways are significantly enriched in platelets after indirect co-culture with CTC41, CTC41.4, and CTC41.5G cells vs*.* control. Venn diagrams show the shared enriched transcripts in each experimental condition. The heatmaps **(C–F)** of the common transcripts among the three sample groups illustrate their expression level (red, high; blue, low) compared with control. *Abbreviations*: PLT = Platelets, NES = Normalized enrichment score, FDR = False discovery rate.

We next investigated by RNA-seq the platelet transcriptome changes upon indirect culture (presence of a Transwell insert) with the three CTC lines for 24 h. We used transcriptome data from freshly isolated platelets from the same donor as control to eliminate alterations due to the time impact (24 h-culture) and not CTC influence. Volcano plots highlighted the differential expression of many RNAs in co-cultured platelets compared with control (platelets alone) (Figure 2A) (Supplementary Tables S1–S6). The Venn diagram showed that indirect co-culture with each CTC line led to a specific expression profile and that only 24 transcripts were differentially regulated in all three conditions **(** Figure 2B). Among these 24 transcripts, the count numbers of AC132872.5, AL031282.2, Ras-related protein 37 (*RAB37*), myristoylated alanine-rich protein kinase C substrate (*MARCKS*), cerebellin 4 precursor (*CBLN4*), *CD38*, alpha-1,3-mannosyl-glycoprotein 4-beta-N-acetylglucosaminyltransferase B (*MGAT4B*), DENN domain containing 6B (*DENND6B*), glycoprotein IX platelet (*GP9*), TNF receptor-associated factor 1 (*TRAF1*), deltex E3 ubiquitin ligase 1 (*DTX1*), tubulin beta 4B class IVb (*TUBB4B*), atypical chemokine receptor 3 (*ACKR3*), triggering receptor expressed on myeloid cells like 1 (*TREML1*), TNF receptor superfamily member 4 (*TNFRSF4*), whirlin (*WHRN*), AP003068.2, transglutaminase 2 (*TGM2*), ribonuclease A family member 1, pancreatic (*RNASE1*), and prostaglandin E synthase (*PTGES*) transcripts were significantly enhanced. Chitinase acidic (*CHIA*), C-C motif chemokine ligand 24 (*CCL24*), myogenesis regulating glycosidase (*MYORG*) and C-C motif chemokine receptor 2 (*CCR2*) transcript count numbers were decreased**.** Moreover, among the significant transcripts, *PTGES* expression was increased upon indirect co-culture with the three CTC lines [CTC41 (log FC = 3.920256295, *p =* 0.0005*,* CTC41.4 (Log FC = 3.268002339 *p = 0.0026*), and CTC41.5G (Log FC = 3.034793859, *p = 0.0013*)] compared with control. PTGES is involved in modulating vascular tone and platelet reactivity (Friedman, Ogletree, Haddad and Boutaud, 2015).

GSEA revealed that RNAs were significantly enriched in genes related to EMT, hypoxia, inflammatory response, and apoptosis. A Venn diagram showed for each of these pathways, the transcripts that were enriched in all three experimental conditions (i.e., platelets indirectly co-cultured with the CTC41, CTC41.4, and CTC41.5G line). For instance, vascular endothelial growth factor A (*VEGFA*), tropomyosin 4 (*TPM4*), secreted protein acidic and cysteine rich (*SPARC*), secreted phosphoprotein 1 (*SPP1*), and transforming growth factor beta 1 (*TGFß1*) were significantly enriched in the EMT pathway in all three experimental conditions [CTC41 (*p =* 0.021)*,* CTC41.4 (*p = 0.013*), and CTC41.5G (*p = 0.012*)] (Figure 2C). In the hypoxic pathway, 29 mRNAs were significantly upregulated in all three experimental conditions compared with control, including phosphoglycerate kinase 1 (*PGK1*), enolase 1 (*ENO1*), enolase 2 (*ENO2*), lactate dehydrogenase A (*LDHA*), BTG anti-proliferation factor 1 (*BTG1*) and TNF alpha induced protein 3 (*TNFAIP3*) (Figure 2D). Interleukin 2 receptor subunit beta (*IL2RB*), interleukin 1 beta (*IL1B*), and interleukin 4 receptor (*IL4R*) were among the shared genes significantly enriched in the inflammatory response pathway, whereas perforin 1 (*PRF1*), glutathione peroxidase 4 (*GPX4*), and spermidine/spermine N1-acetyltransferase 1 (*SAT1*) were shared differentially expressed genes in the apoptotic pathway (Figures 2E, F).

In addition, platelets exposed to the CTC lines showed significantly enriched RNAs in genes related to coagulation. RNAs related to the PI3K-AKT-mTOR signaling pathway were enriched in platelets exposed to the CTC 41.4 line, and RNAs related to TNFα signaling via NF-κB and mTORC1 signaling in platelets exposed to the CTC41.5G line.

It is widely reported that mediators like CTCs can influence the RNA repertoire of platelets. Platelets are highly dynamic cells that can communicate and influence their environment and are involved in various steps of cancer development and progression by supporting tumor growth, survival, and dissemination. Reciprocally, cancer cells can directly and/or indirectly influence platelet RNA content, resulting in their “education”. These direct and indirect tumor cell-platelet interactions can activate intracellular signaling pathways in both cell types to support consecutive steps of the metastatic cascade. We observed significant enrichment in genes related to important pathways in metastasis formation, such as EMT, hypoxia, inflammatory response, apoptosis, coagulation, PI3K-AKT-mTOR, TNFα, and mTORC1 signaling, after co-culturing platelets with the different CTC lines.

### 3.3 CTC gene expression changes upon direct co-culture with platelets

**FIGURE 3:**
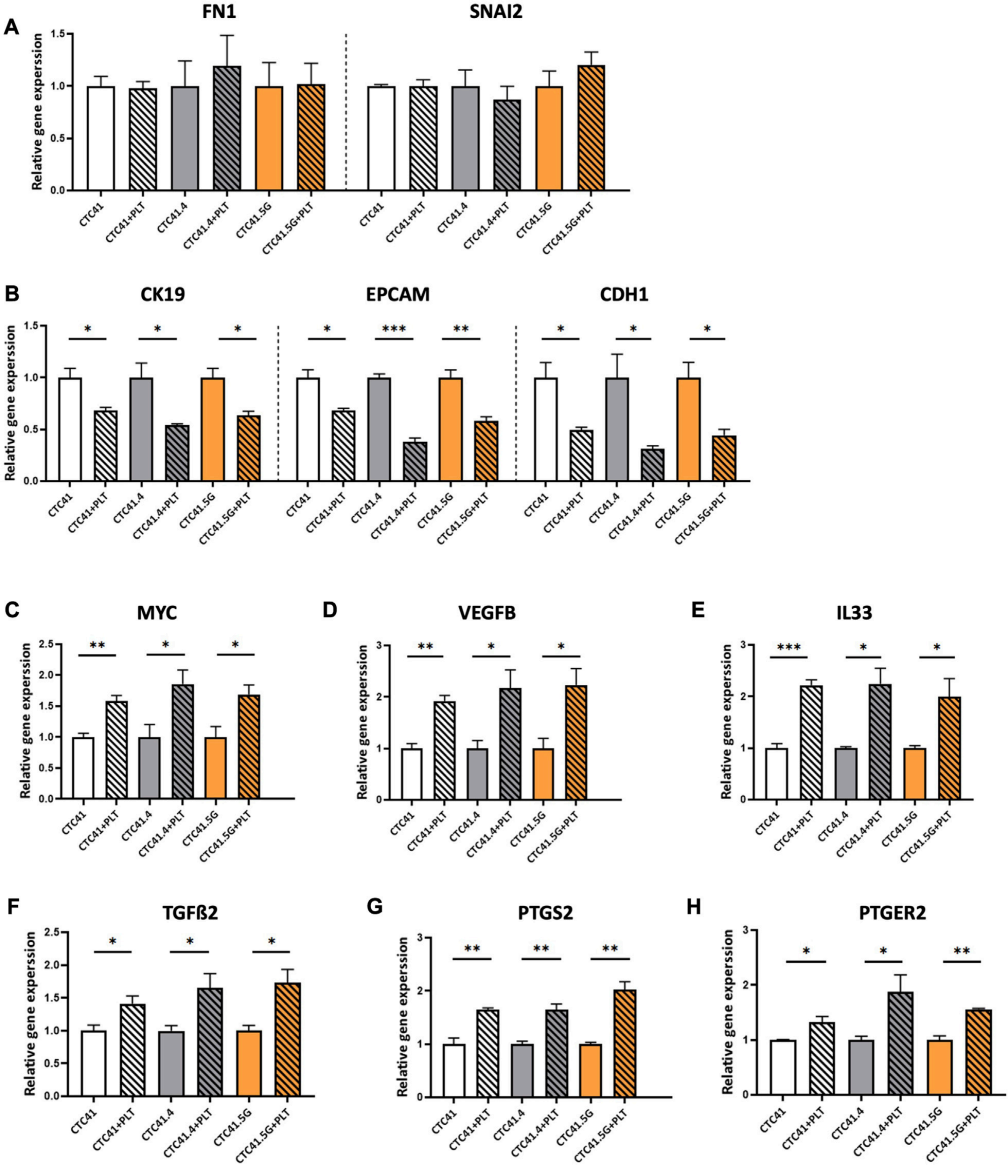
RT-qPCR analysis of **(A)** *FN1* and *SNAL2*, **(B)** *CK19*, *EPCAM* and *CDH1*, **(C)** *MYC*, **(D)** *VEGFB*, **(E)** *IL33*, **(F)** *TGFß2*, **(G)** *PTGS2*, and **(H)** *PTGER2* in CTC lines co-incubated with platelets (striped bars) compared with control (CTCs alone; without stripes); expression in control was set to 1; values are the mean ± SEM, *n* = 3/group; *p < 0.05, **p < 0.005, ***p < 0.0005 (unpaired t-test). Abbreviations: PLT = platelets, *FN1* = fibronectin, *CK19* = cytokeratin 19, EPCAM = epithelial cell adhesion molecule, *CDH1* = E-cadherin, *MYC* = MYC proto-oncogene, *VEGFB* = vascular endothelial growth factor B, *IL33* = interleukin 33, *TGFß2* = transforming growth factor beta 2, *PTGS2* = prostaglandin-endoperoxide synthase 2, *PTGER2* = prostaglandin E receptor 2.

Various steps in the metastatic cascade (i.e., immune surveillance escape, EMT, adhesion to the vascular endothelium, trans-endothelial migration and neoangiogenesis) are closely influenced by platelet-tumor cell interactions (Gay and Felding-Habermann, 2011; Guo, Cui, Pei and Xu, 2019; Ishikawa et al., 2016; Labelle, Begum and Hynes, 2011b; Labelle et al., 2014; Labelle and Hynes, 2012; Leblanc and Peyruchaud, 2016; Marcolino et al., 2020; X. Wang, Zhao, Wang and Gao, 2022). Concerning EMT, the work by Labelle and coworkers highlighted a new metastasis-promoting effect of platelets*.* They showed that the number of lung metastasis foci dramatically increases when tumor cells are incubated with platelets. This suggests that the interaction between tumor cells and platelets directly supports tumor metastasis (Labelle et al., 2011b). Moreover, tumor cells co-incubated with platelets showed morphological modifications and increased invasiveness. Specifically, the epithelial markers E-cadherin and claudin 1 were downregulated in platelet-treated cancer cells, whereas snail, vimentin, fibrin, and plasminogen activator inhibitor-1 were upregulated. Moreover, N-cadherin relocated from the cell-cell interface to the cytoplasm, indicating that tumor cells underwent platelet-induced EMT (Labelle et al., 2011b). Several studies confirmed platelet role in enhancing the mesenchymal features of cancer cells through direct or indirect cross-talk mediated by the release, of instance, of TGF-β (Labelle et al., 2011b; Guo et al., 2019; Zhang et al., 2019; Karolczak and Watala, 2021). In colorectal cancer, the cancer cell-platelet interaction activates the Wnt-β-catenin pathway, leading to an aggressive EMT phenotype (Lam et al., 2017). To verify this finding in our CTC lines and to address platelet-induced EMT in CTCs, we tested the expression level of genes involved in EMT by RT-qPCR in CTCs indirectly co-cultured with platelets. Co-culture with platelets did not affect the expression of snail family transcriptional repressor 2 (*SNAI2*) and fibronectin 1 (*FN1*), two mesenchymal markers, whereas it significantly decreased the expression of epithelial markers, such as CK19 (*CK19*) (CTC41 vs*.* CTC41+PLT: *p = 0.0283*; CTC41.4 vs*.* CTC41.4+PLT: *p = 0.0302*; CTC41.5G vs*.* CTC41.5G + PLT: *p = 0.020875*), epithelial cell adhesion molecule (*EPCAM*) (CTC41 vs*.* CTC41+PLT: *p = 0.0192*; CTC41.4 vs*.* CTC41.4+PLT: *p = 0.0002*; CTC41.5G vs*.* CTC41.5G + PLT: *p = 0.0074*), and E-cadherin (*CDH1*) (CTC41 vs*.* CTC41+PLT: *p = 0.026*; CTC41.4 vs*.* CTC41.4+PLT: *p = 0.0378*; CTC41.5G vs*.* CTC41.5G + PLT: *p = 0.0241*) (Figures 3A, B). Previous studies showed that in cancer cell lines co-cultured with platelets, the expression of epithelial markers is decreased (as observed also in CTCs), and the expression of transcription factors and mesenchymal markers is increased (whereas in CTCs it remained stable). Spillane et al. investigated the induction by platelets of a mesenchymal-like phenotype in eleven epithelial cancer cells. They observed a decrease in the expression of the epithelial markers CDH1 and EpCAM in approximately 50% of the selected cell lines. They showed that platelet adhesion to cancer cells appears to be a universal process, resulting in an EMT-driven phenotype. Interestingly, they also proposed an EMT model that supports the lack of change in mesenchymal markers once cells have already gone through EMT (Spillane et al., 2021). Similarly, we recently showed in CTCs that epithelial and mesenchymal markers are co-expressed to facilitate the cell ability to go back and forth between cellular states (Balcik-Ercin, Cayrefourcq, Soundararajan, Mani and Alix-Panabières, 2021). These data suggest that CTCs in the bloodstream have already acquired a partial EMT state. Consequently, although CTCs showed slightly less epithelial features after co-incubation with platelets, their mesenchymal features could not be further enhanced. Flow cytometry data confirmed the reduced expression of EpCAM and CK-19, two epithelial markers (Supplementary Figure S2).

Additionally, a transcriptomic analysis of CTC lines compared with primary and metastatic colon cancer lines showed differential expression of genes implicated in cancer development and progression (Alix-Panabières et al., 2017). Therefore, to investigate whether platelets could influence the expression of these genes, we evaluated by RT-qPCR the expression of *MYC,* vascular endothelial growth factor B (*VEGFB*) and interleukin 33 (*IL33*). The MYC oncogene is a transcription factor that controls 15% of all genes, including those involved in protein synthesis, cell metabolism, cell cycle, and survival (Ahmadi, Rahimi, Zarandi, Chegeni and Safa, 2021). MYC is overexpressed in breast, lung, colon, pancreas, and prostate cancer (C. Wang et al., 2021). Its overexpression has been correlated with lower progression-free survival and overall survival, and plays a role in resistance to therapy in colorectal cancer (Strippoli et al., 2020; Sun et al., 2020). *VEGFB* is implicated in angiogenesis and tumor progression (Hicklin and Ellis, 2005) and *IL33* is a prognostic marker of cancer progression and angiogenesis (Choi et al., 2009; Cui et al., 2018; Shen, Liu and Zhang, 2018). All three genes were upregulated in the three CTC lines co-cultured with platelets compared with controls [*MYC*: CTC41 vs*.* CTC41+PLT (*p = 0.006*), CTC41.4 vs*.* CTC41.4+PLT (*p = 0.047*), and CTC41.5G vs. CTC41.5G + PLT (*p = 0.040*); *VEGFB*: CTC41 vs*.* CTC41+PLT (*p = 0.003*), CTC41.4 vs*.* CTC41.4+PLT (*p = 0.038*), and CTC41.5G vs. CTC41.5G + PLT (*p = 0.030*); and *IL33*: CTC41 vs*.* CTC41+PLT (*p = 0.000*), CTC41.4 vs*.* CTC41.4+PLT (*p = 0.015*), and CTC41.5G vs. CTC41.5G + PLT (*p = 0.047*)] (Figures 3C–E). *MYC* upregulation was in line with a previous study showing that platelets can influence MYC regulation and consequently cell proliferation (Mitrugno et al., 2017). Moreover, *TGFß2* gene expression was increased in all three lines co-cultured with platelets (CTC41 vs. CTC41+PLT: *p* = 0.045; CTC41.4 vs. CTC41.4+PLT: *p* = 0.046; and CTC41.5G vs. CTC41.5G + PLT: *p* = 0.029) (Figure 3F). Similarly, in patients with colorectal cancer, *TGFß2* is upregulated (Qu, Chen, Zhang and He, 2021).

Lastly, we found that co-incubation of the three CTC lines with platelets significantly increased the expression of prostaglandin-endoperoxide synthase 2 (*PTGS2*) (CTC41 vs*.* CTC41+PLT: *p = 0.006*; CTC41.4 vs*.* CTC41.4+PLT: *p = 0.006*; CTC41.5G vs. CTC41.5G + PLT: *p = 0.002*) and prostaglandin E receptor 2 (*PTGER2*) (CTC41 vs*.* CTC41+PLT: *p = 0.037*; CTC41.4 vs*.* CTC41.4+PLT: *p = 0.047*; CTC41.5G vs*.* CTC41.5G + PLT: *p = 0.002*) (Figures 3G, H). Previous studies showed that platelets contribute to regulate PTGS2 (COX-2) expression in cancer cells (Dovizio et al., 2015) and that *COX-2* overexpression is a hallmark of tumor invasion, metastasis, and poor prognosis in colorectal cancer and other cancer types (Singh, Berry, Shoher, Ramakrishnan and Lucci, 2005; Szweda, Rychlik, Babińska and Pomianowski, 2019; Sheng et al., 2020; Kolawole and Kashfi, 2022). Moreover, *PTGS2* overexpression is an early event in colorectal cancer development (Vogel et al., 2014), and *PTGER2* plays a critical role in PTGS2-induced colon cancer development in an experimental system (Baba et al., 2010).

## 4 Discussion

In the last few years, an increasing number of studies emphasized the role of platelets in carcinogenesis (Gay and Felding-Habermann, 2011). Complex bidirectional interactions between tumor cells and platelets seem to be crucial for cancer development and cohesive aggregates of CTCs are considered to have increased invasive potential (Sarioglu et al., 2015). This involves direct (cell-cell interactions) and indirect interactions (with the release of proteins or other biomolecules) and also the formation of tumor-platelet aggregates (D’Ambrosi et al., 2021).

These interactions lead to changes in both cancer cells and platelets. For instance, Plantureux et al. (2020) explored the interaction of platelets and colorectal cancer cells and found that it leads to the production of extracellular vesicles that inhibit tumor growth, but promote metastasis. Specifically, this interaction induced platelet spreading, the release of their granule content, and the generation of three types of microparticles that express platelet markers and/or tumor markers. Nilsson et al. (2011) brought evidence of the uptake of tumor-derived RNA by platelets. This was followed by the development of a platform for the analysis of the tumor-educated platelet transcriptome as a companion cancer diagnostic tool in the context of personalized medicine (Best et al., 2015). Most recently, the platelet-educated tumor concept was introduced showing that platelets can influence cancer cells through the highly efficient transfer of lipids, proteins, and RNA via different mechanisms (direct contact, internalization, or extracellular vesicles) (Rodriguez-Martinez et al., 2022). Importantly, platelets are anucleate cells that lose their RNA and have a short life span in the blood circulation (Angénieux et al., 2016; Mills, Green and Ingolia, 2017). Therefore, variations in their RNA content is likely to be the result of an effect on their progenitors (megakaryocytes) (Davizon-Castillo et al., 2020). However, the underlying mechanism is unclear. Additionally, a recent clinical trial (NCT02453139) revealed platelet cloaking in patients with metastatic prostate cancer, and a connection between CTC and white cell numbers (Brady et al., 2020), highlighting the clinical significance of platelet-CTC interactions. Long-term administration of aspirin, which reduces platelet aggregation and activation, inhibits the COX-1/TXA2 pathway in platelets and consequently, decreases platelet aggregation on tumor cells, endothelial activation, tumor cell adhesion to the endothelium, and recruitment of metastasis-promoting monocytes/macrophages and ultimately the formation of a pre-metastatic niche (Lucotti et al., 2019).

Despite the many studies on the cross-talk between cancer cells and platelets *in vitro*, *in vivo*, and in humans (Labelle et al., 2011b; Cho et al., 2012; Orellana et al., 2015; Hu et al., 2017), to the best of our knowledge, no study investigated *in vitro* the CTC-platelet interaction. This lack of data may be related to CTC rarity in the peripheral blood but also to the huge challenge of performing functional studies on CTCs and platelets in culture. Indeed, permanent CTC lines, compared to the CTC pool in blood, are unique biological material because they can give rise to distant metastases. Moreover, they allow obtaining huge amounts of CTCs because they can be amplified *in vitro*. In this study, we asked how healthy platelets that come into contact with metastasis-competent CTCs are altered/educated by these very aggressive tumor cells. It would be interesting to study the CTC-platelet interaction at different stages of cancer, but this is not easy to address because CTCs usually die after enrichment if they do not have stemness properties. Here, we used three permanent colon CTC lines (Cayrefourcq et al., 2015; Soler et al., 2018) and co-culture conditions to mimic the proximity and cross-talk between CTCs and platelets in order to determine whether they reciprocally influence their transcriptome landscape.

We first incubated platelets in CTC conditioned medium. After 24 h of exposure, platelets were “activated” faster than when exposed to control medium. The “activation” of platelets was first suggested by the formation of clusters or clots, as observed upon incubation with thrombin, a key factor in the coagulation cascade (Sang, Roest, de Laat, de Groot and Huskens, 2021). The formation of platelet aggregates around tumor cells may support their survival and may protect them from immune eradication (Gay and Felding-Habermann, 2011; Schmied, Höglund and Meinke, 2021). This interaction enhances the adhesion of tumor cells to the endothelium. This leads to tumor cell arrest and facilitates their extravasation to potentially reach a distant organ (Anvari, Osei and Maftoon, 2021). Then, we confirmed platelet activation by measuring the percentage of platelets that express CD41/61 and CD62P, two markers associated with activation. We found that the percentages of platelets expressing these markers were increased.

Then, we assessed changes at the transcriptomic level in platelets co-cultured with the three CTC lines (but separated by a Transwell insert). In this condition, CTCs and platelets are not in direct contact, but platelets can be affected by the CTC secretome. We observed significant differential mRNA expression in co-cultured platelets compared with control (platelets alone). Moreover, the Venn diagram demonstrated that platelets exposed to each CTC line had a specific expression profile, and only 24 differentially expressed transcripts were shared. Differentially expressed transcripts were mainly associated with the hypoxia, inflammatory response, apoptosis and EMT pathways. The enrichment of specific pathways, such as hypoxia and apoptosis, might be explained by the RNA exchange between CTCs and platelets. Interestingly, in addition to the shared differentially expressed transcripts in platelets exposed to the three CTC lines, platelets exposed to each CTC line presented a unique signature. For instance, differentially expressed transcripts were related to coagulation in platelets exposed to CTC 41 cells, to the PI3K-AKT-mTOR signaling pathway in platelets exposed to CTC 41.4 L cells, and to TNFα signaling via NF-κB and mTORC1 signaling in platelets exposed to CTC41.5G cells. However, in our study, we did not explore whether RNA cargo differences in platelets were due to nucleic acid uptake or release. Interactions with CTCs might 1) limit the degradation of specific RNAs or 2) promote the transfer of such RNAs into platelets. In *vitro* conditions, the transfer of RNAs is unlikely following the activation of platelets when they are in contact with CTCs, thus leading to the release (or degradation) of RNAs. Moreover, as platelets are anucleate cell fragments, *de novo* transcription regulation is impossible. Therefore, the presence of more RNA fragments or sequences cannot be understood as increased gene expression. All changes in platelet RNA content are due to pre-mRNA splicing and the translational machinery. Although indirect co-culture with CTCs affected the RNA level in platelets, some of these changes could be explained also by the fact that some RNA sequences are resistant to degradation during platelet activation. Additionally, the enrichment of circular RNA in platelets and not in their progenitors (megakaryocytes) implicates the degradation of linear RNAs. It also suggests that circulating platelets lose >90% of their progenitor mRNAs and that translation in platelets occurs against the backdrop of a highly degraded transcriptome (Alhasan et al., 2016).

**FIGURE 4:**
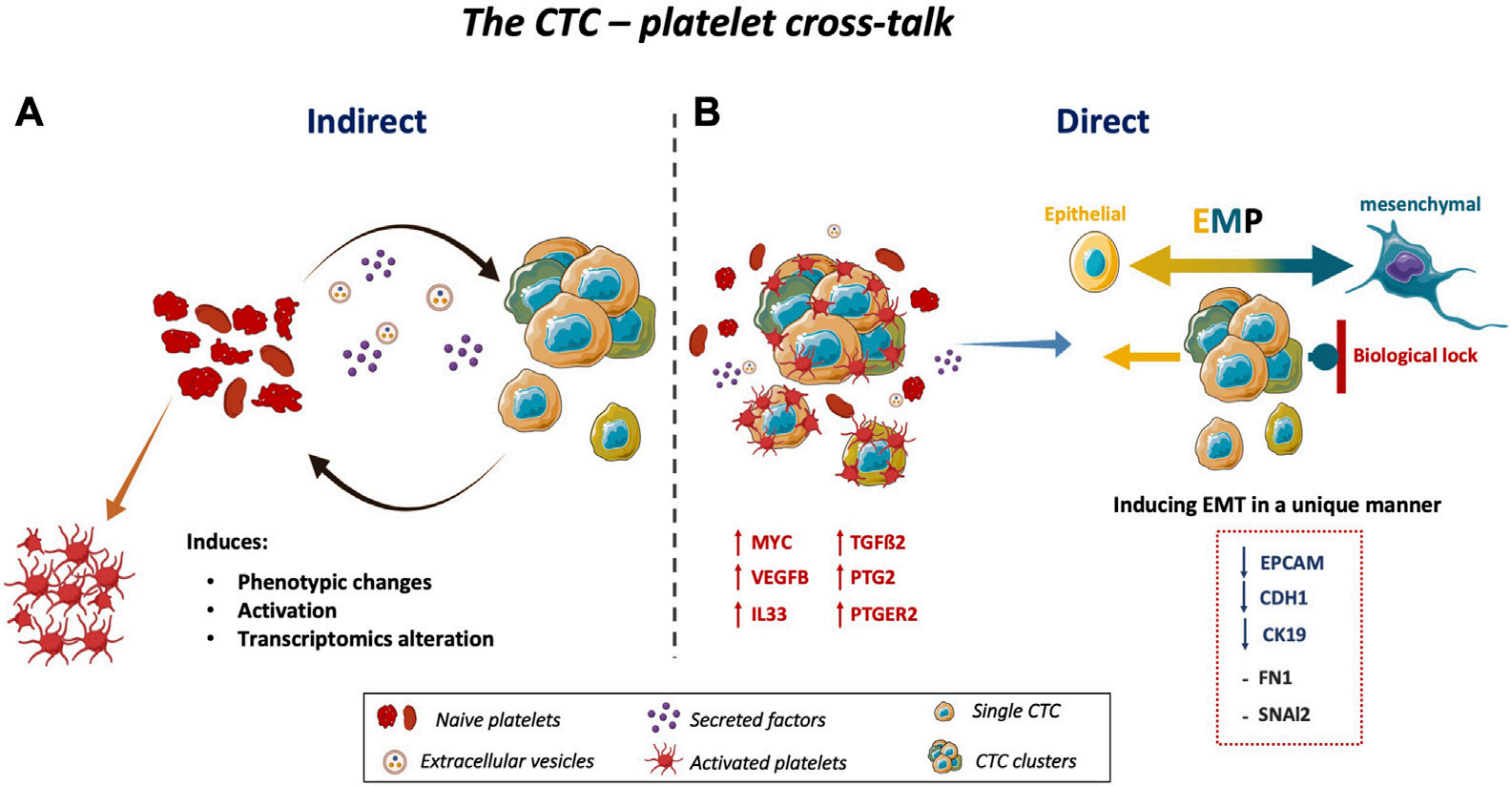
Schematic representation of the CTC-platelet cross-talk. **(A)** CTC-platelet interactions cause phenotypic changes, activation, and transcriptome alternation in platelets, and also **(B)** increase the invasive properties of CTCs by upregulating *MYC*, *VEGFB*, *IL33*, *PTGS2*, and *PTGE*R2 as well as *TGFß2* in CTCs. Co-culturing CTCs with platelets induces or maintains the EMT state of CTCs. These cells have a major epithelial phenotype with some features of invasive cells. Upon EMT induction by interacting with platelets, the expression of epithelial markers was decreased, without upregulation of mesenchymal markers, in the three colon CTC lines. This might be explained by a specific biological lock to maintain CTCs in a partial EMT, allowing them to undergo MET immediately. This plasticity, called epithelial-to-mesenchymal plasticity, gives to CTCs a more aggressive phenotype and facilitates their quick adaptation in their new tumor environment to initiate a new tumor at a distant site. *Abbreviations*: EMT = epithelial-to-mesenchymal transition, MET = mesenchymal-to-epithelial transition, EMP = epithelial-to-mesenchymal plasticity; *TGFß2* = transforming growth factor beta 2, *MYC* = MYC proto-oncogene, *VEGFB* = vascular endothelial growth factor B, *IL33* = interleukin33, *PTGS2* = prostaglandin-endoperoxide synthase 2, *PTGER2* = prostaglandin E receptor 2.

Moreover, direct (no Transwell insert) co-culture with platelets affected gene expression in the three colon CTC lines. The expression of *TGFß2* was upregulated, whereas the expression of epithelial markers was reduced. It should be kept in mind that these CTC clones were selected during cancer progression and treatment pressure and show heterogeneity and plasticity. They underwent partial EMT and acquired some mesenchymal features, while maintaining epithelial characteristics (i.e., EMT state) (Williams, Gao, Redfern and Thompson, 2019). This hybrid state allows CTC to revert to the epithelial state (i.e., mesenchymal-epithelial transition) (Bocci, Jolly and Onuchic, 2019). Although it has been reported that platelets increase mesenchymal features in cancer cell lines (Labelle et al., 2011a), we did not observe this effect in our CTC lines. This might be due to the existence of one or more biological locks that prevent CTCs from becoming fully mesenchymal cells, but allow them to acquire epithelial-to-mesenchymal plasticity (Chin and Lim, 2019; Balcik-Ercin et al., 2021). Consequently, CTCs that undergo partial EMT express epithelial and mesenchymal markers and this increases their survival fitness to successfully finalize the metastatic cascade. Indeed, CTCs display a more aggressive phenotype than pure epithelial cancer cells and can rapidly adapt (mesenchymal-epithelial transition) to initiate a new tumor in a new environment, at a distant site from the primary tumor (Balcik-Ercin et al., 2021). Moreover, platelets altered the expression of genes involved in cancer development. Altogether, these data highlight the key role of platelets in promoting and maintaining a more aggressive CTC subpopulation that can give rise to distant metastases (Figure 4). This interaction and the impact on CTC epithelial features by platelets might also influence CTC isolation and characterization as a liquid biopsy biomarker. Indeed, the CellSearch^®^ system, the only United States Food and Drug Administration (FDA) approved system for CTC analysis, is an epithelial marker-based technology to detect clinically relevant CTCs. However, many CTCs in patients with different cancer types (Fehm et al., 2010; Gervasoni et al., 2011; Krebs et al., 2012) do not have sufficient epithelial characteristics to allow their detection with this tool. CTC interactions with platelets during their journey in the circulation lead to a reduction of CTC epithelial features and influence their phenotypic plasticity. The adhesion of platelets to CTCs also provides a ubiquitous surface marker for CTC isolation. Platelet-targeted isolation is generally applicable to CTCs with epithelial and mesenchymal phenotypes, thus facilitating the characterization of all CTC clones. Currently, platelet-cloaked CTCs might not be captured by conventional positive and negative isolation mechanisms. Yet, their characterization may uncover important biological information on blood-borne metastases and cancer immunology (Jiang et al., 2017).

Lastly, we should acknowledge some limitations. First, the amount of RNA recovered from platelets was often limited due to the delicate nature of platelets and clot formation in the culture plates. Moreover, we did not investigate the factors released by the colon CTC lines. Second, we used platelets from healthy donors, and studies have already shown that platelets from different healthy donors have distinct RNA profiles, which could have affected our results (Supernat et al., 2021). Third, the physical effects of cancer cells on platelets should be addressed in follow-up studies. Fourth, the direct co-culture of CTCs with platelets invariably leads to CTC decoration by platelets. The RNA content is lower in platelets than CTCs (2.2 fg vs. 10–50 pg) (M. G. Best, Vancura and Wurdinger, 2017), and the pipetting, washing, and centrifugation steps eliminate most platelets; however, the expression analysis of CTC-platelet co-cultures might include both platelet- and CTC-derived transcripts. Therefore, ideally, the same set of genes in resting and activated platelets should be analyzed to exclude their influence on the final results. Moreover, we performed the platelet transcriptomic analysis using platelets cultured indirectly with CTCs and not in direct contact. This limitation is due to technical issues of physically separating platelets attached to CTCs that did not allow us to analyze platelets that directly interact with CTCs. Finally, we used an *in vitro* culture system, that is, very different from the *in vivo* conditions (systemic circulation) characterized by the presence of shear forces and many other cell types to interact with, including immune cells (Liu et al., 2023).

In conclusion, to our best knowledge, this is the first work that explored the functional cross-talk between CTCs and platelets. We found that in contact with the CTC secretome, platelet activation was increased and transcripts supporting CTC dissemination were upregulated. The platelet-CTC cross-talk in the bloodstream might promote CTC survival and increase their chance to reach the right distant organ for metastasis formation. Our *in vitro* study showed that platelets and CTCs reciprocally interact. Moreover, platelets contribute to CTC plasticity (partial EMT state). However, more studies are required to evaluate *in vivo* the exact mechanisms involved in this cross-talk. In addition, analysis of the CTC and platelet secretome and proteome may improve our understanding of the pathways leading to tumor-educated platelets and highly aggressive CTCs.

## Data Availability

The datasets presented in this study can be found in online repositories. The names of the repository/repositories and accession number(s) can be found below: https://www.ncbi.nlm.nih.gov/geo/query/acc.cgi?acc=GSE230557.
