# Supplementary figures and images for: In vitro cross-talk between metastasis-competent circulating tumor cells and platelets in colon cancer: a malicious association during the harsh journey in the blood
Source: Front Cell Dev Biol. 2023 Aug 2;11:1209846. doi: 10.3389/fcell.2023.1209846 (PMC10433913; doi:10.3389/fcell.2023.1209846)

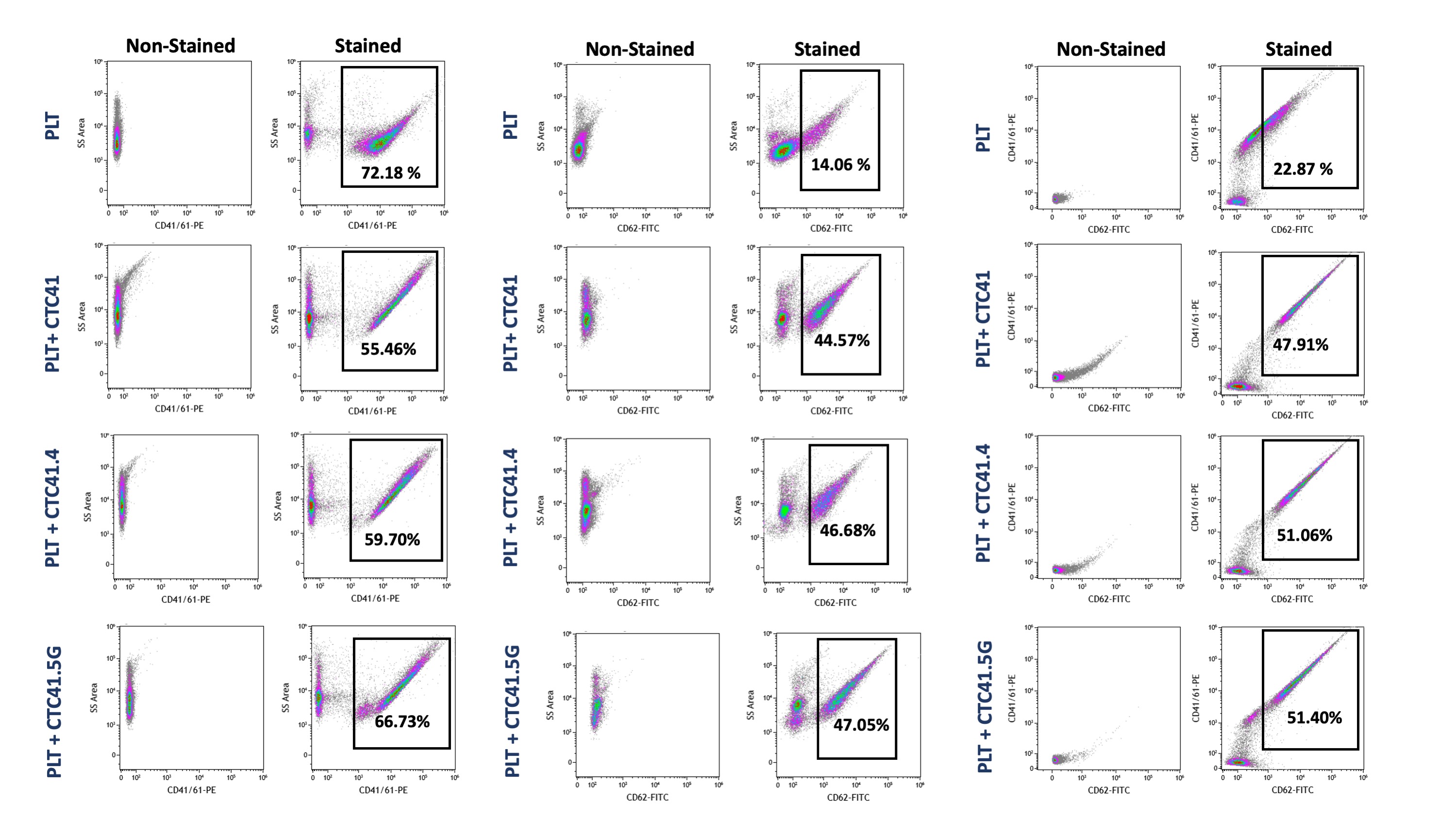

Supplement: Supplementary file 3 [file Image1.JPEG]

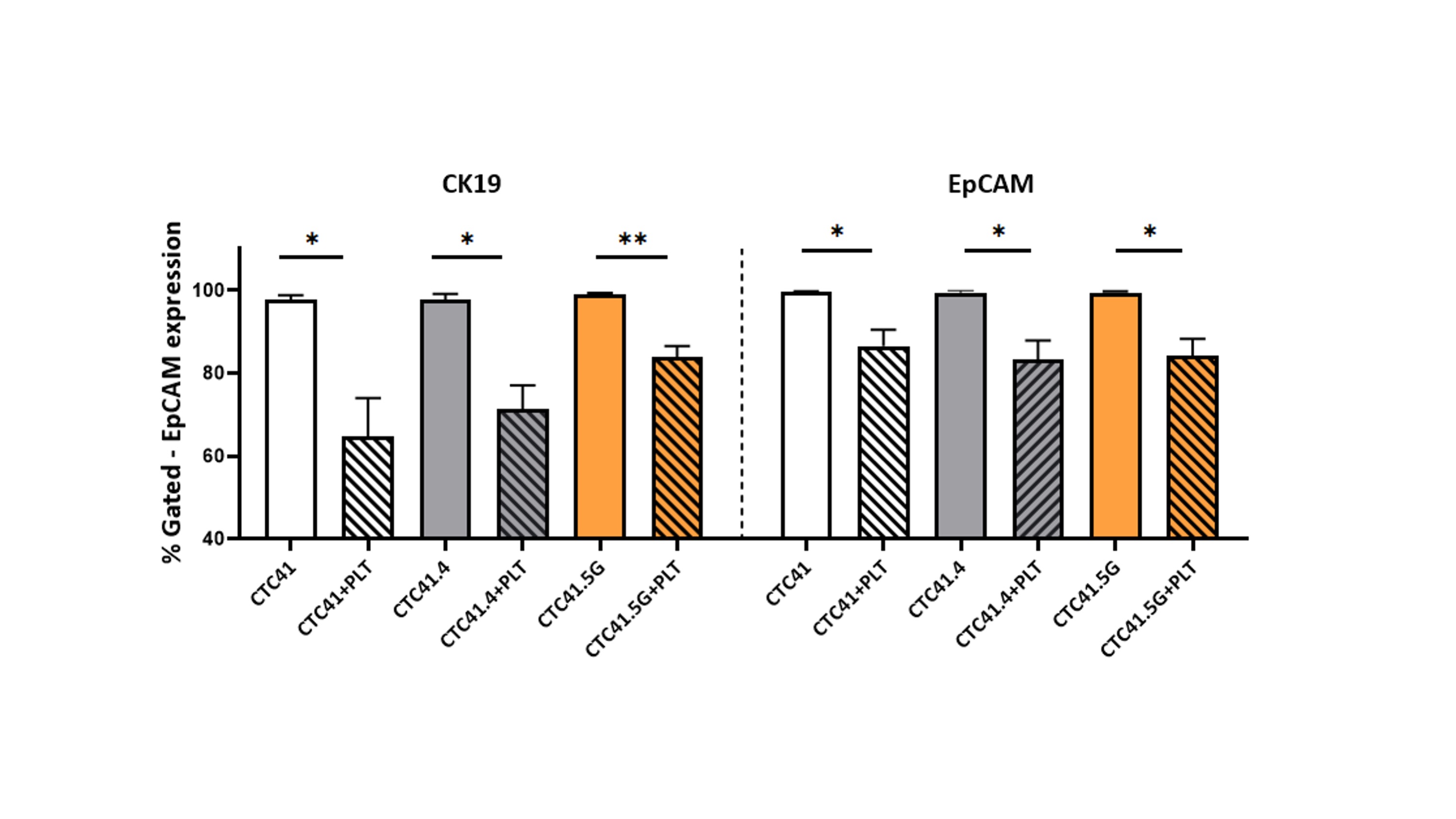

Supplement: Supplementary file 4 [file Image2.JPEG]
