# Supplementary material for: In vitro cross-talk between metastasis-competent circulating tumor cells and platelets in colon cancer: a malicious association during the harsh journey in the blood
Source: Front Cell Dev Biol. 2023 Aug 2;11:1209846. doi: 10.3389/fcell.2023.1209846 (PMC10433913; doi:10.3389/fcell.2023.1209846)
Supplement: Supplementary file 9 [file DataSheet1.DOCX]

**Supplementary Figures**

| **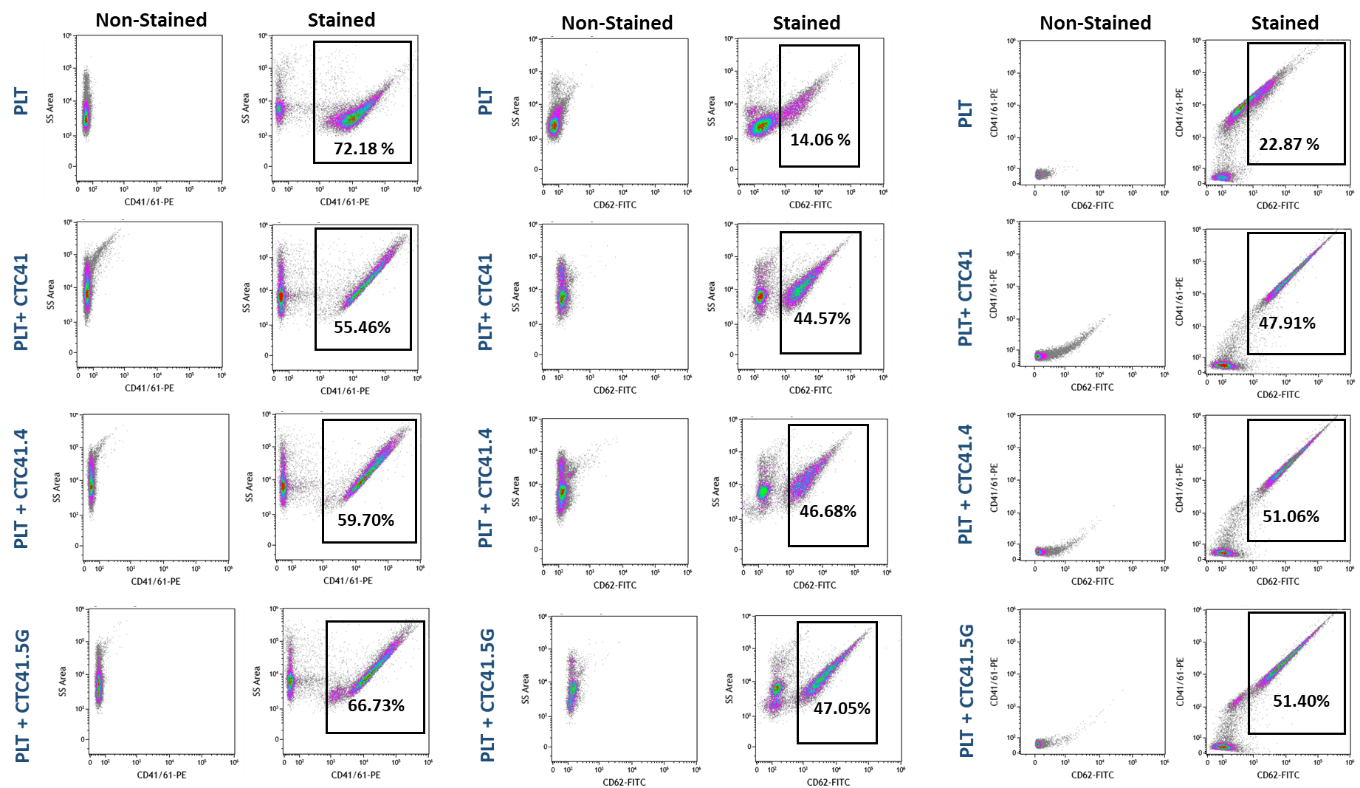** |
| --- |
| \| **Supplementary Figure 1:** Flow cytometry gating strategy for the detection of CD41/61^+^, CD62^+^ and CD41/61^+^CD62^+^ platelets incubated with CTC41, CTC41.4 and CTC41.5G conditioned medium compared with control (normal medium; PLT), n = 3/group. Gates for positive signals were designed for each marker based on control (PLT). *Abbreviations*: PLT = Platelets. \| \| --- \| |

| **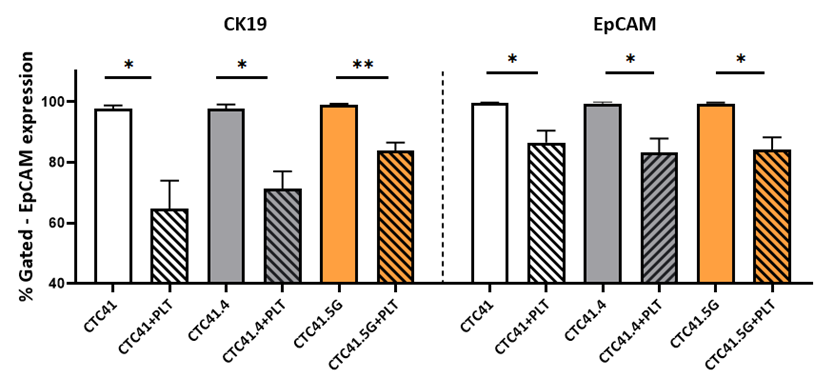** |
| --- |
| **Supplementary Figure 2:** Flow cytometry analysis of CK19 and EpCAM expression in CTC41, CTC41.4, and CTC41.5G cells co-cultured with platelets (striped bars) compared with control (CTCs alone; without stripes), n = 3/group; *p <0.05, **p <0.005, ***p <0.0005 (unpaired *t*-test). *Abbreviations*: PLT = Platelets, CK19 = cytokeratin 19, EpCAM = epithelial cell adhesion molecule. |
